# A novel partitivirus conferring hypovirulence by affecting vesicle transport in the fungus *Colletotrichum*

**DOI:** 10.1128/mbio.02530-23

**Published:** 2024-01-09

**Authors:** Jun Zi Zhu, Ze Lan Qiu, Bi Da Gao, Xiao Gang Li, Jie Zhong

**Affiliations:** 1 Hunan Provincial Key Laboratory for Biology and Control of Plant Diseases and Insect Pests, Hunan Agricultural University, Changsha, Hunan, China; National Institutes of Health, Bethesda, Maryland, USA

**Keywords:** mycovirus, hypovirulence, *Colletotrichum *species, *Partitivirus*, endocytosis

## Abstract

*Colletotrichum* spp. are economically important phytopathogenic fungi that cause anthracnose in a variety of plant species worldwide. Hypovirulence-associated mycoviruses provide new options for the biological control of plant fungal diseases. Here, we found a novel partitivirus from *Colletotrichum alienum* and named it Colletotrichum alienum partitivirus 1 (CaPV1). CaPV1 contained two dsRNA segments encoding an RNA-dependent RNA polymerase and a capsid protein and was classified under the genus *Gammapartitivirus* of the family *Partitiviridae*. CaPV1 significantly decreased host virulence, mycelial growth, appressorial development, and appressorium turgor but increased conidial production with abnormal morphology. In addition, CaPV1 could be successfully transfected into other *Colletotrichum* species, including *C. fructicola*, *C. spaethianum*, and *C. gloeosporioides,* and caused hypovirulence, indicating the broad application potential of this virus. CaPV1 caused significant transcriptional rewiring of the host fungus *C. alienum*. Notably, some genes related to vesicle transport in the CaPV1-infected strain were downregulated, consistent with the impaired endocytosis pathway in this fungus. When the Rab gene CaRab7, which is associated with endocytosis in vesicle transport, was knocked out, the virulence of the mutants was reduced. Overall, our findings demonstrated that CaPV1 has the potential to control anthracnose caused by *Colletotrichum*, and the mechanism by which *Colletotrichum* induces hypovirulence is caused by affecting vesicle transport.

**IMPORTANCE:**

*Colletotrichum* is a kind of economically important phytopathogenic fungi that cause anthracnose disease in a variety of plant species worldwide. We found a novel mycovirus of the *Gammapartitiviru*s genus and *Partitiviridae* family from the phytopathogenic fungus *Colletotrichum alienum* and named it CaPV1. This study revealed that CaPV1 infection significantly decreased host virulence and fitness by affecting mycelial growth, appressorial development, and appressorium turgor. In addition, CaPV1 could also infect other *Colletotrichum* species, including *C. fructicola*, *C. spaethianum*, and *C. gloeosporioides*, by viral particle transfection and resulting in hypovirulence of these *Colletotrichum* species. Transcriptomic analysis showed that CaPV1 caused significant transcriptional rewiring of the host fungus *C. alienum*, especially the genes involved in vesicle transport. Moreover, endocytosis and gene knockout assays demonstrated that the mechanism underlying CaPV1-induced hypovirulence is, at least in part, caused by affecting the vesicle transport of the host fungus. This study provided insights into the mechanisms underlying the pathogenesis of *Colletotrichum* species and mycovirus-fungus interactions, linking the role of mycovirus and fungus vesicle transport systems in shaping fungal pathogenicity.

## INTRODUCTION

*Colletotrichum* spp. are among the most economically important phytopathogenic fungi that cause anthracnose disease in a variety of plant species worldwide (1). Based on their scientific and economic importance, *Colletotrichum* spp. have been ranked as the eighth most important plant pathogenic fungus in the world (1, 2) and have also been used as models to study the infection mechanism of hemiotrophic pathogenic fungi (3). *Colletotrichum alienum* was first reported as the pathogen fungus on mango tissues in Mexico (4). In recent years, *C. alienum* was reported as the causal organism of anthracnose disease on *Aquilaria sinersis* (5) and postharvest anthracnose of mango in China (6). Finding efficient and environmentally friendly methods to control crop anthracnose disease is of great theoretical and practical significance for reducing chemical pesticide application, environmental pollution and the development of pathogen resistance.

Mycoviruses (or fungal viruses) are viruses infecting almost all major taxa of fungi, including plant-pathogenic fungi and yeasts (7–10). Since they were first reported in the cultivated button mushroom *Agaricus bisporus* in 1962, knowledge of mycoviruses has expanded exponentially during the past several decades (9). Mycoviruses have been attracting an increasing amount of attention not only because some mycoviruses confer hypovirulence to their host but also because they enhance our understanding of virus diversity and evolution (10, 11). Some mycoviruses with the ability to cause phenotypic alterations resulting in hypovirulence and debilitation in the host are considered to be useful biological control agents. The successful use of Cryphonectria parasitica hypovirus 1 (CHV1), which is associated with hypovirulence to control chestnut blight in some parts of Europe, has provided a new method for the biological control of fungal plant diseases (9). By contrast, some mycoviruses have beneficial effects. For example, some mycoviruses can confer hypervirulence, including enhanced growth, aggressiveness, virulence, and sporulation (12–17), and confer competitive ability in some yeasts containing L-A virus and the killer satellite dsRNAs (18, 19). Furthermore, some mycoviruses might play important roles in complex mycoviruses-fungi or mycoviruses-fungi-plants mutualistic symbiosis, such as the mycovirus inhabiting the endophytic fungus *Curvularia protuberata,* which could enhance the heat tolerance of both the host fungus and the associated plant (20). In addition, some mycoviruses might participate in the interactions between biocontrol fungi and their host, such as mycoviruses in *Trichoderma* spp. (21), *Metarhizium majus* (22), and *Beauveria bassiana* (15). In addition, some mycoviruses can regulate the metabolite production, multistress tolerance, and drug resistance of their host fungi (11, 23, 24). Overall, although most mycoviruses have no recognizable effect on their host fungi, they might have some still unexplored roles in complex symbiosis, at least from an ecological perspective (9).

Viruses in the *Partitiviridae* family are divided into five recognized genera: *Alphapartitivirus*, *Betapartitivirus*, *Cryspovirus*, *Deltapartitivirus*, and *Gammapartitivirus*, as well as two proposed genera, namely, Epsilonpartitivirus and Zetapartitivirus (25–27). The genome of this family members consists of two linear dsRNA molecules ranging in length from 1.4 to 2.4 kbp, each containing a single large ORF. The larger segment (dsRNA1) encodes the RNA-dependent RNA polymerase (RdRp), and the smaller segment (dsRNA2) encodes the capsid protein (CP) (25, 26). These two genome segments are packaged into spherical virus particles with diameters of approximately 25–40 nm.

Vesicle trafficking is a conserved mechanism underlying intracellular transport and affects the physiology and pathogenicity of filamentous fungi (28). Some secretory pathways direct proteins and lipids to the plasma membrane by vesicle trafficking through the endoplasmic reticulum (ER) and the Golgi apparatus (29). Proteins recruited by a specific Rab, so-called effectors, possess diverse functions in the vesicle trafficking system (30). Among these proteins, Rab7 is a conserved protein required in the late endocytic pathway and in lysosome biogenesis (31). Previous studies showed that protease p29 of CHV1 was associated with vesicle membranes and behaves as an integral membrane protein of the vesicular fraction derived from the fungal *trans*-Golgi network (TGN) (32). A novel +ssRNA mycovirus, namely Colletotrichum fructicola RNA virus 1 (CfRV1), also appeared to be a capsidless virus and potentially associated with vesicles within fungal cells, as visualized by transmission electron microscopy (33). However, the relationship between mycovirus-associated hypovirulence and the fungal vesicle trafficking system is still not clear.

To date, several dsRNA and +ssRNA mycoviruses from different families have been identified and characterized in diverse *Colletotrichum* species (34–43). Among these, only a few mycoviruses, such as a polymycovirus CcFV1 in *C. camelliae* (39), an epsilonpartitivirus ClPV1 in *C. liriopes* (43), and a chrysovirus CfCV1 in *C. fructicola* (40), have been reported to cause hypovirulence. However, the number of mycoviruses isolated from *Colletotrichum* species is still limited, and their biological and molecular traits are still not well understood. In this study, a novel mycovirus named Colletotrichum alienum partitivirus 1 (CaPV1) was isolated and characterized from the plant pathogenic fungus *C. alienum*. We showed that CaPV1 was a potential virocontrol agent since it could reduce the virulence of *C. alienum*, spread to and lead to hypovirulence of other *Colletotrichum* species, including *C. fructicola*, *C. spaethianum,* and *C. gloeosporioides*. Interestingly, we found that CaPV1 caused hypovirulence by transcriptional reprogramming of the fungus, especially by influencing the vesicle trafficking system. The objective of this research was to identify and elucidate the impact and mechanism of the mycovirus CaPV1 in *Colletotrichum* species, aiming to establish a theoretical foundation for the biocontrol of *Colletotrichum* anthracnose.

## RESULTS

### Identification and characterization of a novel mycovirus in *C. alienum*

**Fig 1:**
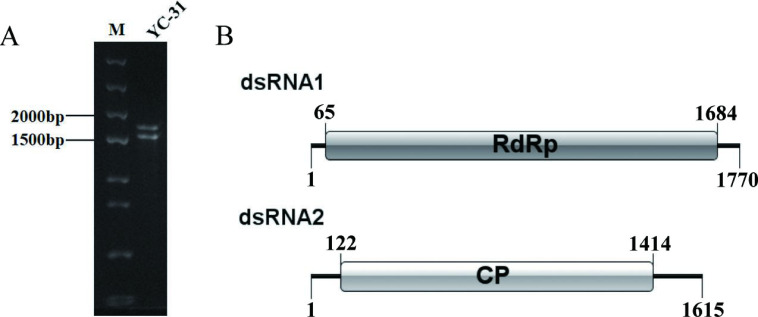
Agarose gel electrophoresis analysis and putative genomic organization of virus CaPV1. (**A**) Electrophoretic profile of dsRNA preparations extracted from *Colletotrichum alienum* strain YC-31 following treatment with DNase I and S1 nuclease. Lanes 1 and 2 indicate a DNA Marker of 2,000 bp and dsRNA of CaPV1. (**B**) Schematic representation of the genome organization of CaPV1. The long boxes represent open reading frames (ORFs) encoding the putative RNA-dependent RNA polymerase (RdRp) and capsid protein (CP) of CaPV1.

DsRNAs were extracted from the mycelia of *C. alienum* strain YC-31. After digestion with DNase I and S1 nuclease and examination by 1% agarose gel electrophoresis (Fig. 1A), two distinct dsRNA bands (termed dsRNA 1 and dsRNA 2) approximately 1.8 and 1.6 kbp in size were observed, thus suggesting the presence of potential mycovirus in strain YC-31.

Following RT-PCR with tagged random primers and rapid cDNA end amplification, the full-length cDNA sequences of dsRNA 1 and dsRNA 2 were determined to be 1,770 and 1,651 bp, respectively. dsRNA 1 and dsRNA 2 were similar to the RNA-dependent RNA polymerase (RdRp) and capsid protein (CP) genes of *partitiviruses*, respectively. Thus, the two dsRNA segments might represent the genome of a putative partitivirus, which we named CaPV1. The cDNA sequences of each of the two dsRNA segments were predicted to include a single ORF, which were named ORF1 and ORF2. The 5′ and 3′-untranslated regions (UTRs) were 64 and 86 bp in dsRNA 1 and 121 and 201 bp in dsRNA 2, respectively. The putative genome organization of CaPV1 is shown in Fig. 1B. The full-length cDNA sequences for dsRNA 1 and dsRNA 2 were deposited in GenBank with the accession numbers OR266096 and OR266097, respectively.

ORF1 of CaPV1 was predicted to encode a polypeptide comprising 539 aa residues with a calculated molecular mass of 62.8 kDa. A BLASTp search revealed that the 62.8 kDa protein was similar to the RdRp of partitiviruses, including Metarhizium brunneum partitivirus 2 (MbPV2, accession no.: QTC11257.1, query coverage: 100%; E value: 0; identity: 88.31%) and Fusarium mangiferae partitivirus 2 (FmPV2, accession no.: UBZ25878.1, query coverage: 100%; E value: 0; identity: 85.90%). Conserved domain analysis revealed that the ORF1-encoded protein contained an RdRp domain (RdRp_1; pfam00680) possessing six conserved motifs that are characteristic of dsRNA viruses in the family *Partitiviridae*. ORF2 in dsRNA 2 potentially encoded a 430 aa protein with a molecular mass of 46.7 kDa. A homology search revealed that it was similar to the CP of partitiviruses, such as Colletotrichum partitivirus 1 (accession no.: ALD89089.1; E-value: 0; query cover: 100%; identity: 70.85%).

**Fig 2:**
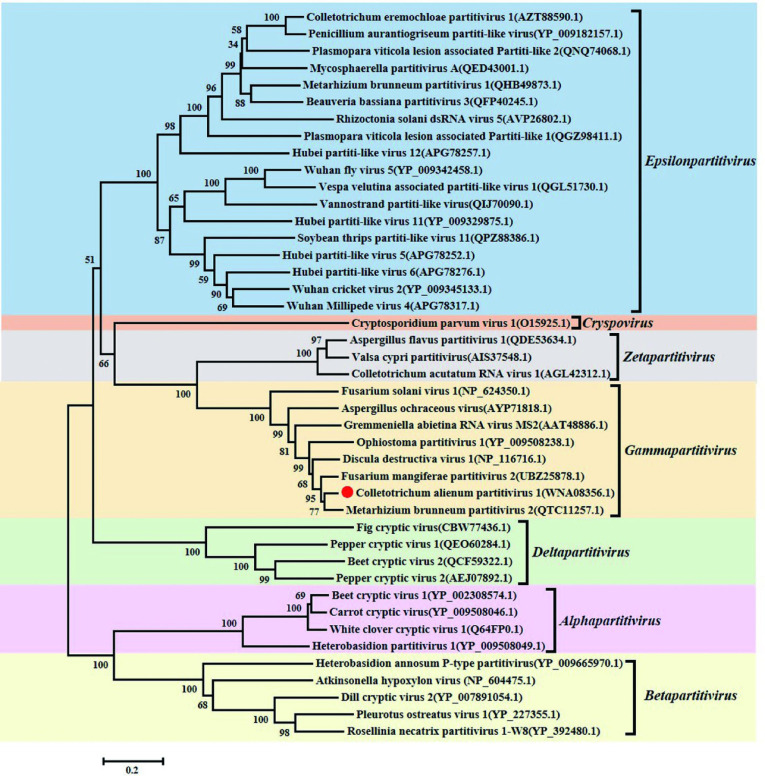
Phylogenetic analysis of CaPV1 based on RdRp. The numbers in the parentheses indicate the GenBank accession number of amino acid sequences used for phylogenetic tree construction. The notes at the branch points indicate the bootstrap values supporting the branches. The scale bars indicate the estimated number of substitutions per 100 amino acids.

Phylogenetic analyses were conducted based on the protein sequences of RdRp and CP. The RdRp-based phylogenetic tree showed that CaPV1 was most closely related to the RdRp of members of the genus *Gammapartitivirus* in the family *Partitiviridae*, including MbPV2 (Fig. 2). Phylogenetic analysis with the CP also clustered CaPV1 with Gammapartitiviruses, indicating a similar taxonomic status in the family *Partitiviridae* (Fig. 1). Since the threshold criteria for the distinction of species were 90% and 80% similarity for RdRp and CP, respectively (44), we considered CaPV1 to be a new member of the genus *Gammapartitivirus* in the family *Partitiviridae*.

### Purification and characterization of viral particles

**Fig 3:**
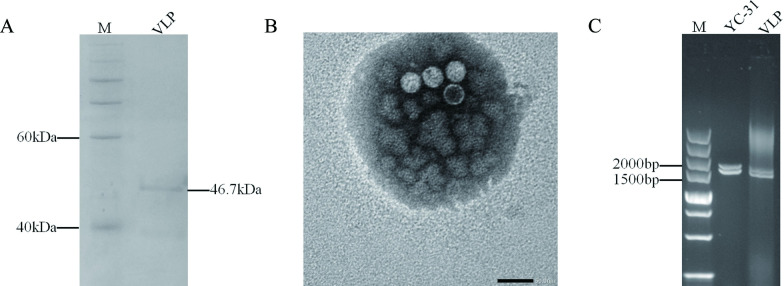
Virus particles isolated from the mycelia of CaPV1-infected strain YC-31 and analysis of its protein and nucleic acids. (**A**) SDS-PAGE electrophoresis (12%) of the purified virus particles showing the protein band of the coat protein. (**B**) Isometric viral particles with diameters of 30–40 nm were observed by transmission electron microscopy. (**C**) Agarose gel electrophoresis of dsRNA extracted from the mycelia of YC-31 and viral particles.

Virus particles of CaPV1 were purified from the mycelia of strain YC-31 by sucrose density gradient centrifugation. SDS-PAGE electrophoresis indicated an approximately 46 kDa single protein band, which was consistent with the predicted molecular mass of the ORF2-encoded protein (Fig. 3A). Under transmission electron microscope (TEM) examination, isometric, nonenveloped, spherical viral particles of 30–40 nm in diameter, that are characteristic of members of the *Partitiviridae* family, were observed (Fig. 3B). In addition, the dsRNA of CaPV1 was also extracted from the purified viral particles, exhibiting the same size as those purified from the mycelia of strain YC-31 (Fig. 3C).

### CaPV1 significantly decreases fungal virulence

**Fig 4:**
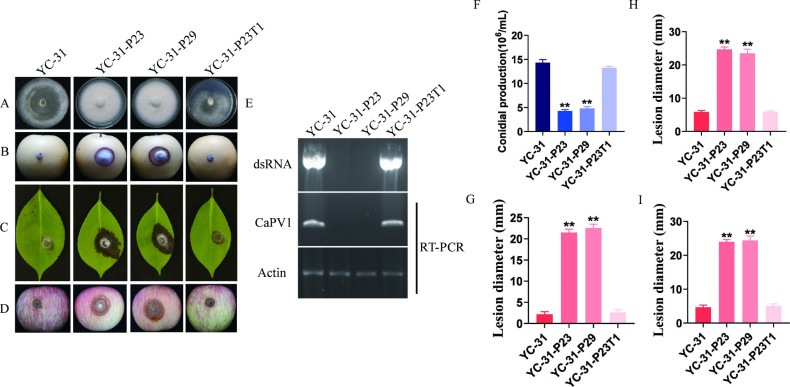
Biological function analysis of CaPV1 infection. Strain YC-31 was infected with CaPV1, while YC-31-p23 and YC-31–29 were virus-free derivative strains of YC-31 obtained by protoplast regeneration. YC-31-P29T1 was the derivative strain of YC-31-P29 infected by CaPV1 via protoplast transfection. (**A**) Colony morphology of strains YC-31, YC-31-P23, YC-31-P29, and YC-31-P23T1 cultured on PDA for 5 days. (**B, G**) Pathogenicity tests and lesion sizes of strains YC-31, YC-31-P23, YC-31-P29, and YC-31-P23T1 on pear. (**C, H**) Pathogenicity tests and lesion sizes of camellia leaves. (**D, I**) Pathogenicity tests and lesion sizes on apples. (**F**) Conidial production of strains YC-31, YC-31-P23, YC-31-P29, and YC-31-P23T1. (**E**) Detection of CaPV1 in strains YC-31, YC-31-P23, YC-31-P29, and YC-31-P23-T1 using dsRNA extraction and RT‒PCR methods, with the actin gene of *C. alienum* serving as an internal control. Statistical analysis was performed for comparisons between different strains using Student’s *t*-test. **, *P* < 0.01.

To elucidate the effects of CaPV1 in *C. alienum*, we conducted protoplast regeneration experiments for virus curing. Two virus-free isolates, YC-31-P23 and YC-31-P29, were obtained and confirmed by dsRNA extraction and RT-PCR detection (Fig. 4A and E). Furthermore, protoplasts of isolate YC-31-P23 were transfected with the viral particles of CaPV1 using PEG-mediated protoplast transfection. A derivative virus-transfected strain designated YC-31-P23T1 was randomly selected and verified for CaPV1 infection (Fig. 4A and E) and then selected for further analysis.

Compared with virus-free strains YC-31-P23 and YC-31-P29, the CaPV1-infected strains YC-31 and YC-31-P23T1 were significantly different, as they showed more melanin accumulation and sparser mycelia on the colonies (Fig. 4A). Virulence assays on detached pear fruits, camellia leaves, and apple fruits were conducted (Fig. 4B to D). The average lesion areas caused by YC-31-P23 and YC-31-P29 were significantly larger than those caused by YC-31 and YC-31-P23T1, indicating that the virulence of isolates YC-31 and YC-31-P23T1 was reduced (Fig. 4H through I). Overall, the virus-curing and transfection assays provided direct evidence that CaPV1 was associated with hypovirulence in its fungal host.

### CaPV1 infection influences fungal growth, conidial production, and morphology

**Fig 5:**
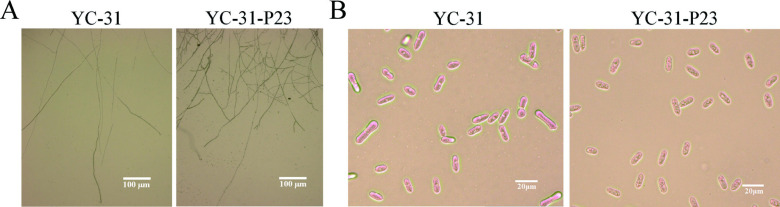
Microscopic observation of mycelia (**A**) and conidia (**B**) morphology of strains YC-31 and YC-31-P23. Scale bars of the mycelia and conidia were 100 µm and 20 µm, respectively.

Hyphal tip and conidial morphology were observed by light microscopy. Compared with those of strain YC-31-P23, the hyphal tips of strain YC-31 were sparse and had fewer branches (Fig. 5A). Conidia and appressoria both play important roles in the pathogenesis of *Colletotrichum* (45). Notably, the virus-infected strain YC-31 had a higher conidia production compared to that of the virus-free strain YC-31-P23 (1.38 × 10^7^ /mL vs 4.63 × 10^6^ /mL) (Fig. 4F). The conidia of strain YC-31-P23 were cylindrical and elliptic, with blunt round or slightly pointed ends, and were 7.5–15.2 × 3.5–6.4 µm in size [average size = (12.04 ± 0.28) μm × (4.83 ± 0.08) μm, *n* = 50]. By contrast, abnormally long linear conidia were found in strain YC-31 (Fig. 5B), which had a size of 10.1–26.7 × 3.9–8.9 µm [average size = (15.92 ± 0.65) μm × (5.81 ± 0.15) μm, *n* = 50].

### CaPV1 modulates appressorium formation and appressorium turgor

**Fig 6:**
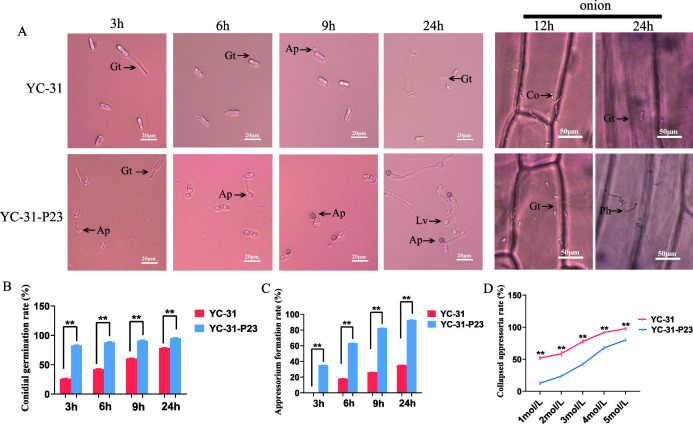
Observation and calculation of conidial germination, appressorium formation, and appressorium collapse of strains YC-31 and YC-31-P23. (**A**) Conidial germination and appressorial formation of YC-31 and YC-31-P23 in hydrophobic surfaces and onion epidermal cells. Co, conidia. Gt, germ tube. Ap, appressorium. Iv, infection vesicle. Ph, primary hyphae. (**B**) Conidial germination rates of YC-31 and YC-31-P23. (**C**) Appressorium formation rates of YC-31 and YC-31-P23. (**D**) Appressorium collapse rates of YC-31 and YC-31-P23. Statistical analysis was performed for comparisons between different strains using Student’s *t*-test. **, *P* < 0.01.

To explore the mechanism underlying the hypovirulence of strain YC-31, we compared conidial germination and appressorial development between the virus-free and virus-infected strains (Fig. 6). After 3 h of cultivation on an artificial hydrophobic surface, the conidial germination rate of strain YC-31-P23 was approximately 83%, which was significantly higher than that of strain YC-31, which had a 26% germination rate. At cultivation for 6 h and 9 h, more than 90% of the conidia of strain YC-31-P23 produced mature appressoria, while less than 27% appressoria formed from the germ tubes of YC-31. After 24 h, appressoria of strain YC-31-P23 were melanized, and some of them formed invasive hyphae. In comparison, strain YC-31 formed relatively longer germ tubes, and most appressoria did not melanize (Fig. 6B and C).

Furthermore, we performed a penetration assay using onion epidermis. Most appressoria of strain YC-31-P23 developed invasive hyphae (also called primary hyphae) and penetrated the onion cells after incubation for 24 h. Although strain YC-31 formed long germ tubes growing on the onion surface, only a few conidia formed appressoria and invasive hyphae (Fig. 6A).

Since strain Yc-31 could still form a small number of appressoria with deficient penetration ability, we further examined the appressorial turgor pressure. The incipient collapse assay showed that the collapse rate in the appressoria of strain YC-31 was significantly higher than that in strain YC-31-P23 (Fig. 6D), suggesting that the turgor pressure of YC-31 was significantly reduced.

### CaPV1 harmed the intracellular morphology of the host fungus

**Fig 7:**
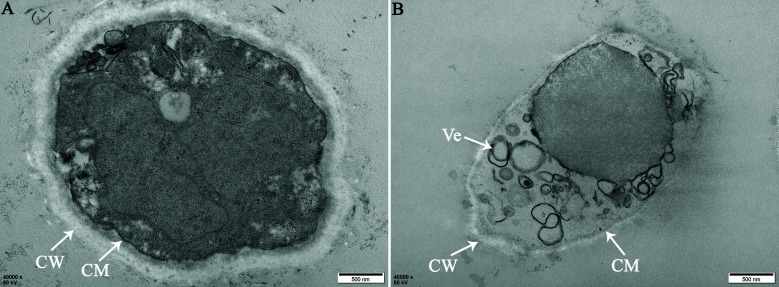
Transmission electron micrographs of ultrathin hyphal sections of strains YC-31 and YC-31-P23. (**A**) Cellular structures were normal, and the cytoplasm was uniform in the virus-free strain YC-31-P23. (**B**) Cell walls were thinning, and protoplasm was heterogeneous and vacuolated, showing giant vesicles inside the hyphal cell of the CaPV1-infected strain YC-31.

TEM observations of thin sections of the CaPV1-infected strain YC-31 and virus-free strain YC-31-P23 were carried out. In YC-31, the cell walls were thinning, and the protoplasm became heterogeneous and vacuolated, with disintegrated organelles. In addition, giant vesicles were present inside the YC-31 hyphae. By contrast, YC-31-P23 appeared normal, with relatively uniform cytoplasm and smooth, thick cell walls. Notably, there were no or significantly fewer similar vesicles in the YC-31-P23 (Fig. 7).

### CaPV1 causes hypovirulence in other *Colletotrichum* species

**Fig 8:**
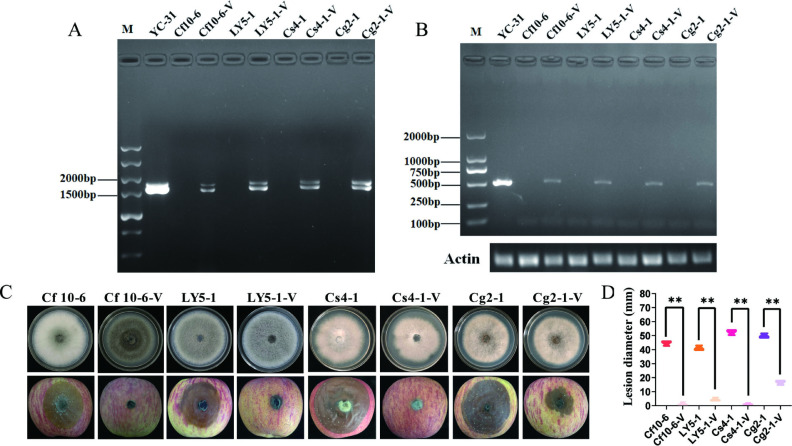
Detection of CaPV1 and comparison of colony morphology and pathogenicity in strains Cf10-6, Cf10-6-V, Cs4-1, Cs4-1-V, LY5-1, LY5-1-V, Cg2-1, and Cg2-1-V. (**A**). Detection of CaPV1 by dsRNA extraction. (**B**) Detection of CaPV1 by RT-PCR. (**C**) Colony morphology and pathogenicity of strains Cf10-6, Cf10-6-V, Cs4-1, Cs4-1-V, LY5-1, LY5-1-V, Cg2-1, and Cg2-1-V. (**C**) Lesion sizes on apples caused by Cf10-6, Cf10-6-V, Cs4-1, Cs4-1-V, LY5-1, LY5-1-V, Cg2-1, and Cg2-1-V. Statistical analysis was performed for comparisons between different strains using Student’s *t*-test. **, *P* < 0.01.

To determine whether CaPV1 could cause hypovirulence in other *Colletotrichum* species, we transfected the purified viral particles of CaPV1 into protoplasts of other *Colletotrichum* species, including *C. fructicola* strains Cf10-6 and LY5-1, *C. spaethianum* strain Cs4-1 and *C. gloeosporioides* strain Cg2-1, using the PEG-mediated protoplast transfection method. Derivative strains Cf10-6-V, LY5-1-V, Cs4-1-V, and Cg2-1-V were obtained from protoplast-regenerated colonies of their respective paternal strains and confirmed to be virus infected by dsRNA extraction and RT-PCR amplification. Biological properties were compared between the Cf10-6-V, LY5-1-V, Cs4-1-V, and Cg2-1-V strains and their corresponding wild-type strains. The results indicated that there were no significant differences in colonial morphology between LY5-1-V, Cs4-1-V, and Cg2-1-V and their respective wild-type strains. However, the colony of Cf10-6-V appeared darker compared to the wild-type strain 10-6. The lesion diameters caused by Cf10-6-V, LY5-1-V, Cs4-1-V, and Cg2-1-V on apple fruits were significantly smaller than those caused by strains Cf10-6, LY5-1, Cs4-1, and Cg2-1, respectively (Fig. 8). Therefore, except for the host fungus *C. alienum*, CaPV1 could also induce hypovirulence in other *Colletotrichum* species, including *C. fructicola*, *C. spaethianum,* and *C. gloeosporioides*.

### CaPV1 infection alters host transcriptome

**Fig 9:**
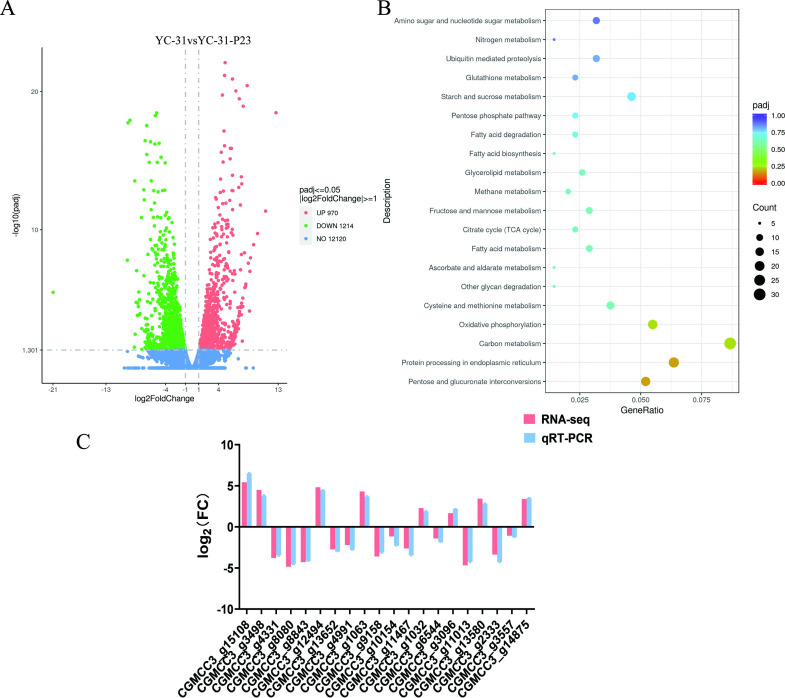
Analysis and verification of differentially expressed genes (DEGs) between strains YC-31 and YC-31-P23. (**A**) Volcano plot of DEGs. (**B**) Bubble diagram of the KEGG enrichment pathway. (**C**) Verification of DEGs between YC-31 and YC-31-P23 transcriptome sequencing data by qRT-PCR. *Colletotrichum fructicola* was used as the reference genome in the transcriptome analysis.

The effects of CaPV1 infection on host transcriptional changes were analyzed by RNA sequencing (RNA-Seq) analysis comparing the virus-infected strain YC-31 and its cured counterpart YC-31-P23. For each sample, three biological replicates were analyzed, and approximately 46–54 million reads for each replicate were obtained. RNA-seq reads were mapped to the reference genome of NCBI GCF_009771025.1 using HISAT2. Transcript abundance was estimated, and differentially expressed genes (DEGs) with a false-discovery rate (FDR) of <0.05 were identified. Final annotation was conducted based on corroborating evidence, including protein sequence similarity and protein domain identification. In comparisons between CaPV1-infected and virus-free strains, a total of 2,184 genes were differentially expressed, with 1,214 genes (55%) downregulated and 970 genes (45%) upregulated in the CaPV1-infected strain (Fig. 9A).

Gene ontology (GO) enrichment analysis showed that most DEGs were related to carbohydrate metabolic processes, transporter activity, transmembrane transporter activity, and iron ion binding (Fig. S2). Therefore, it could be speculated that CaPV1 may regulate the basal metabolism and transport activity of the host fungus.

- Carbohydrate metabolism. The presence of CaPV1 seems to interfere with host carbohydrate metabolism by inhibiting carbohydrate degradation. Among the carbohydrate metabolic processes, 39 of the genes were downregulated, while 30 of the genes were upregulated. Among these genes, those encoding peptidoglycan deacetylase, endopolygalacturonase 1, acetylesterase, etc., were downregulated, possibly indicating the involvement of these genes in many physiological and pathological processes.
- Transporter activity. The infection of CaPV1 increased and decreased the transcription of 26 and 54 genes, respectively, which were predicted to be involved in transporter activity. Several amino acid permeases have provided powerful model systems for addressing the general question of how plasma-membrane transporters are regulated according to environmental changes (46). Therefore, CaPV1 might affect signal transport and conduction between fungal cells.
- Iron ion binding. There were 22 upregulated and 20 downregulated genes related to ion binding due to the presence of CaPV1. The iron ion binding pathway was reported to be closely related to serine metabolism and fungal sporulation in phytopathogenic fungi (47). As inferred through functional classification, these genes may be related to the ion-mediated asexual sporulation of *C. alienum*.

KEGG pathway enrichment analysis revealed that DEGs were mainly enriched in the pathways of carbon metabolism, protein processing in the endoplasmic reticulum, pentose and glucuronate interconversions, and oxidative phosphorylation pathways (Fig. 9B). Overall, the RNA-seq data indicated that significant transcriptional rewiring occurred in the host fungus after infection with the mycovirus CaPV1.

The expression levels of 20 DEGs were further quantified by qRT-PCR. The results showed that there was a relatively high correlation between the RNA-seq data and the qRT-PCR results (Fig. 9C), thus confirming the reliability of the RNA-Seq results.

### CaPV1 infection changes the expression of genes related to vesicle transport

**Fig 10:**
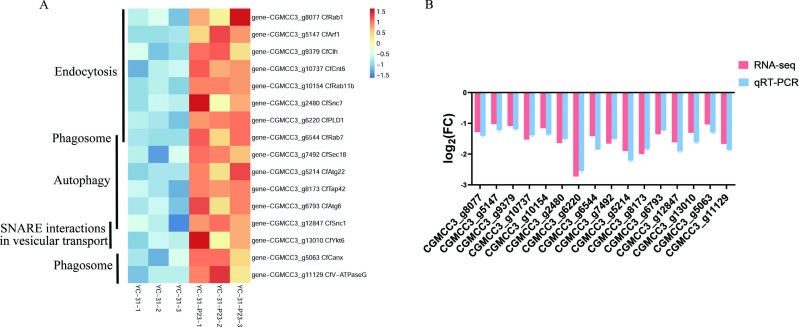
Heatmap and qRT-PCR detection showing the relative expression of selected DEGs related to vesicle transport. (**A**) Heatmap of the selected DEGs. (**B**) qRT-PCR detection of the DEGs. *Colletotrichum fructicola* was used as the reference genome in the transcriptome analysis.

Transcriptome analysis also showed that genes in the pathways of protein processing in the endoplasmic reticulum, ubiquitin-mediated proteolysis, endocytosis, autophagy, SNARE interactions in vesicle transport, and phagosome pathways were also significantly downregulated (Fig. 10A). Representative genes potentially related to vesicle transport were further selected for qRT-PCR detection, and the expression levels of these genes in the CaPV1-infected strain were significantly downregulated compared with those in the virus-free isogenic strain (Fig. 10B). These results suggested that CaPV1 might confer hypovirulence by affecting the host vesicle transport system.

### CaPV1 infection alters host endocytosis

**Fig 11:**
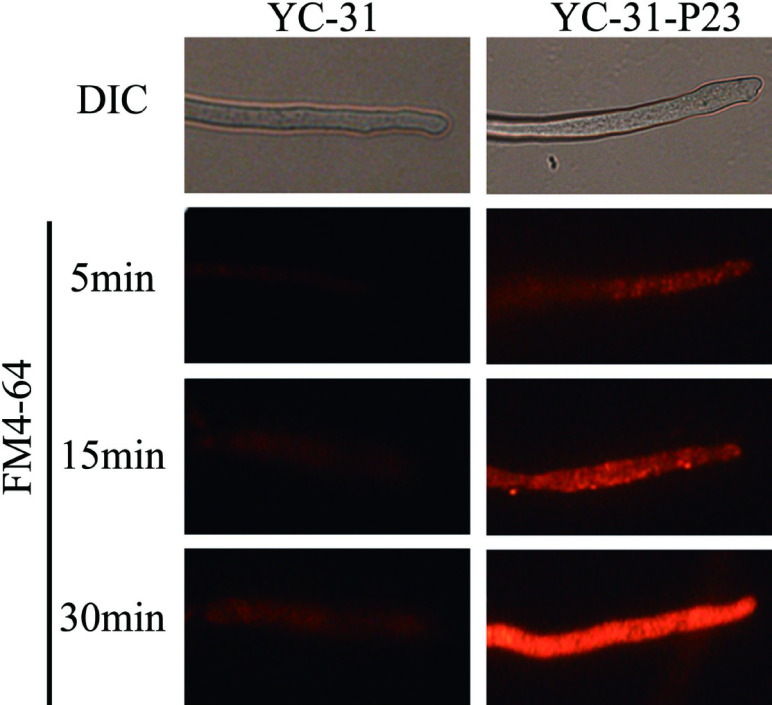
Effect of CaPV1 on endocytosis of host *Colletotrichum alienum* examined by staining with the lipophilic dye FM4-64. Time course images of FM4-64 uptake at the hyphal tips were examined using fluorescence microscopy at time points of 5 min, 15 min, and 30 min. The absorption and internalization of dyes were estimated by observation of red fluorescence.

To examine the effects of CaPV1 infection on host endocytosis, we stained the cells of CaPV1-infected YC-31 and the virus-free strain YC-31-P23 with the lipophilic dye FM4-64. The results showed that the absorption and internalization of dyes in the YC-31 strain were significantly reduced compared with that in strain YC-31-P23. After 5 min of staining, some dye appeared in the cytoplasm of strain YC-31-P23 but was nearly invisible in strain YC-31. At 15 min, the dye was most intense in YC-31-P23, while only a trace of dye existed in strain YC-31. This finding suggested that CaPV1 infection severely affected the endocytosis of its host fungus (Fig. 11).

To investigate whether CaPV1 also influences the endocytosis in other *Colletotrichum* species, we observed the endocytosis in *C. fructicola* strains Cf10-6-V and Cf10-6 by staining with FM4-64. The results revealed that the absorption and internalization of dyes in the hypha of the CaPV1-infected Cf10-6-V strain was significantly weaker compared to strain Cf10-6, even after 15 minutes of staining (Fig. S3). Therefore, CaPV1 also affected the endocytosis in CaPV1-infected *C. fructicola.*

### Functional verification of the key gene *CaRab7* in the endocytosis pathway and vegetative growth and pathogenicity

**Fig 12:**
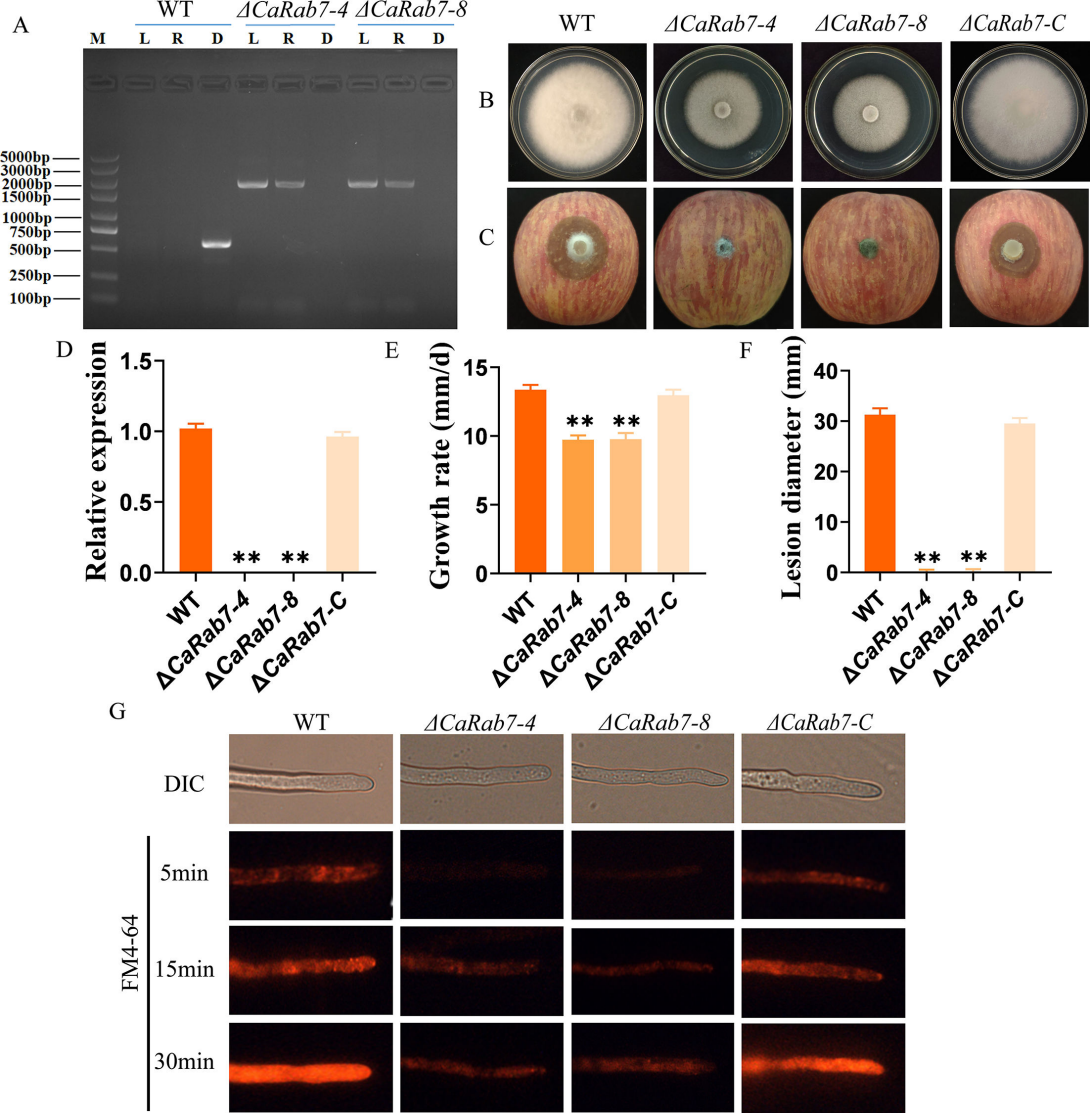
Knockout of the *CaRab7* gene and its biological function analysis. (**A**) PCR validation of the *CaRab7* knockout mutants. M indicates the 5,000 bp DNA marker. L indicates the upstream region of *CaRab7,* and part of the fusion fragment of the *HYG* gene was amplified with the primers LF/HYR. R indicates the downstream region of *CaRab7* and part of the fusion fragment of the *HYG* gene amplified with primers YGF/RR. D indicates that *CaRab7* was amplified with primers CfRab7F/CfRab7R. (**B, E**) Colony morphology and growth rates of *CaRab7* mutants. (**C, F**). Diseased symptoms and lesion diameters of apples that were wounded-inoculated with *CaRab7* mutants and the complemented strain. (**D**) Expression of the *CaRab7* gene in the *CaRab7* mutants by qRT-PCR detection. Statistical analysis was performed for comparisons between different strains using Student’s *t-*test. **, *P* < 0.01. (**G**) Comparison of the endocytosis in mutants Δ*CaRab7-4*, Δ*CaRab7-8*, the wild-type, and complemented Δ*CaRab7-C* strains by FM4-64 staining. Images were captured using fluorescence microscopy at intervals of 5 min, 15 min, and 30 min. The absorption and internalization of dyes were assessed by observing the presence of red fluorescence.

To clarify the function of the DEGs regulated by CaPV1 in the endocytosis pathway, *CaRab7* was selected for genetic manipulation analysis. The gene knockout mutants Δ*CaRab7-4* and Δ*CaRab7-8* were obtained by homologous recombination technology with the PEG-mediated method and PCR and RT-PCR confirmation with three sets of primer pairs (Fig. 12A and D). Moreover, the mutants were also complemented with the wild-type *CaRab7* gene, which restored all defects (Δ*CaRab7-C*). After 5 days of culture on PDA, the growth rates of the mutants Δ*CaRab7-4* and Δ*CaRab7-8* were significantly reduced compared with those of the wild-type and complemented Δ*CaRab7-C* strains (Fig. 12B and E). To characterize the role of *CaRab7* in pathogenicity, mycelial plugs of the wild type, mutants Δ*CaRab7-4* and Δ*CaRab7-8* and complemented Δ*CaRab7-C* were inoculated on wounded apples. After 10 days of inoculation, Δ*CaRab7* mutants showed no lesions compared with the large and typical lesions caused by the WT and Δ*CaRab7-C* strains (Fig. 12C and F). To confirm the role of *CaRab7* in endocytosis, the cells of wild type, mutants *ΔCaRab7-4*, *ΔCaRab7-8*, and complemented Δ*CaRab7-C* were stained by FM4-64. Results showed that the endocytosis in mutants Δ*CaRab7-4* and *ΔCaRab7-8* was also affected, resulting in a substantial reduction in dye absorption and internalization. Overall, these results indicated that *CaRab7* was vital in the endocytosis pathway, which is essential for pathogenicity.

## DISCUSSION

In the present study, we report the discovery and identification of a novel mycovirus, tentatively named CaPV1, from the plant pathogenic fungus *C. alienum*. Partitiviruses generally have a typical genome comprised of two major dsRNA segments and encapsidated with isometric particles, each containing a single ORF (25). The two dsRNA segments of CaPV1 were predicted to encode proteins that shared significant aa identities to the RdRp and CP of viruses in the family *Partitiviridae*. According to genome organization, phylogenetic analysis and viral particle examination, CaPV1 was confirmed to be related to members of the genus *Gammapartitivirus* in the family *Partitiviridae*. To the best of our knowledge, this is the first report of a mycovirus in *C. alienum* fungus. In addition, virus elimination, virus particle transfection, and biological comparison indicated that CaPV1 could lead to hypovirulence of the host *C. alienum* fungus and other *Colletotrichum* species, which might provide a new alternative biocontrol factor for the management of anthracnose disease caused by *Colletotrichum* fungi.

Comparative biological analysis showed that strain YC-31 had less mycelial growth, fewer mycelial tip branches, and a darker colony color, which indicated more pigment synthesis. In *Colletotrichum* spp., the conidia and appressorium play key roles during infection (45). Strain YC-31 produced more conidia than strain YC-31-P23, and some of the conidia were abnormally long. CaPV1, on the one hand, affected host virulence but, on the other hand, can enhance host conidial and pigment production, which is consistent with other previous reports on hypovirulence-associated mycoviruses (48). CaPV1 impacted the conidial germination and appressorium formation rate of strain YC-31. Some conidia were abnormal, and their appressorium could not be melanin pigmentated, and invasive mycelial formation was defective, as observed by an onion epidermal infection test. This finding indicated that CaPV1 caused hypovirulence by affecting the infection ability of the host fungus.

Although most members of the family *Partitiviridae* are generally considered to cause latent infection in their hosts (9), some partitiviruses have been found to induce abnormal morphological and physiological alterations, including hypovirulence (8, 43, 49, 50) or hypervirulence (14). To the best of our knowledge, there have been no reports of hypovirulence caused by partitivirus or even mycovirus infection in the pathogenic fungus *C. alienum*. Pathogenicity assays showed that the virulence of CaPV1-infected strains, such as YC-31 and YC-31-P23T1, was obviously decreased compared to that of virus-free strains, including YC-31-P23 and YC-31-P29. Thus, our study provided evidence that CaPV1 was associated with host hypovirulence. Furthermore, CaPV1 could also infect *C. fructicola*, *C. spaethianum,* and *C. gloeosporioides* and induce hypovirulence by transfecting protoplasts with viral particles, thus extending the host range of CaPV1. These features of CaPV1 point to the anticipated promise of the application of mycoviruses for the biological control of anthracnose disease. However, there are some gaps before CaPV1 could be used as a practical means to control plant diseases, such as improving virus stability during virus proliferation in fungal host, viral transmission through hyphal contact, and discerning the optimum time for virus delivery based on epidemiological studies of anthracnose disease.

RNA sequencing of virus-infected and virus-free strains suggested that the change in host biological phenotype, caused by CaPV1, was partly due to the alteration of host gene expression profiles. Compared to the virus-free strain, the CaPV1-infected host strain YC-31 altered the expression of many genes associated with various biological and metabolic processes related to many basic cellular functions, such as carbon metabolism, pentose and glucuronate interconversions to transporter activity, iron ion binding, protein processing in the endoplasmic reticulum, and oxidative phosphorylation pathways, suggesting that CaPV1 might participate in and influence the synthesis and signal transduction of multiple materials in the host fungus. In previous reports, some mycoviruses in the family *Partitiviridae* causing hypovirulence or hypervirulence could alter their host gene expression in many different pathways. In *Heterobasidion annosum*, infection with HetPV13-an1 *partitivirus* changed the expression of genes related to many basic cellular functions, including carbohydrate metabolism (51). Talaromyces marneffei partitivirus-1 caused aberrant expression of various genes in the host *T. marneffei*, including upregulation of potential virulence factors and suppression of RNA interference (RNAi)-related genes (14). In this study, although the alterations in gene expression might be associated with hypovirulence and other debilitated host phenotypes, the mechanism underlying these effects, especially the gene regulatory network, still requires further investigation.

Notably, multiple signaling pathways related to vesicle transport, such as endocytosis, cell autophagy, and phagosomes, were significantly altered following CaPV1 infection. A number of genes involved in vesicle transport were downregulated in the CaPV1-infected strain. Endocytosis is a fundamental cellular activity utilized by the majority of animal viruses to introduce their genetic material to the cell interior (52). Vesicles in host cells are essential for virus replication and transport by providing targets for virus replication and protection sites for virus transportation (53). In terms of plant viruses, recent studies demonstrated that the southern rice black-streaked dwarf virus (SRBSDV) enters intracellular vesicles in the epithelial cells of its insect vector by engaging VAMP7 and Vti1a proteins in the SNARE complex (54). Although there has been no report of direct interactions between mycoviruses and vesicles in fungal host cells, some reports have suggested a close relationship between mycoviruses and fungal vesicles. CHV1 has been reported to cause the proliferation of vesicles in its *Cryphonectria parasitica* host and to utilize the fungal TGN for replication, which has previously been shown to contain both CHV1 genomic dsRNA and RNA polymerase activity (32). This finding was consistent with later research showing an increased number of vesicles in CHV1-infected strains (55). A similar result was also found in the fungus *C. fructicola*, which was infected by a Hadaka virus, CfRV1, where many irregular large vesicles possessing small vesicles appeared inside the virus-infected fungus, as observed by TEM (33). Some large vesicles that could be fused with the cellular plasmalemma seem to be important components of endocytosis used by other naked viruses (32, 56). In our study, endocytosis of the CaPV1-infected strain was indeed seriously affected, as observed by FM4-64 dye staining. Therefore, we suggest that CaPV1 infection might be associated with vesicle transport. However, to date, the interaction between particle-encapsulated dsRNA virus and the fungal vesicle transport system has rarely been reported. The specific effects and direct interaction targets of CaPV1 in the vesicle transport system still need further investigation.

The vesicle transport system regulating biological processes such as endocytosis and exogenesis is indispensable for the normal growth and development of plant pathogenic fungi (57). Hence, we speculated that CaPV1 might affect host pathogenicity by affecting the host vesicle transport system. Rab family proteins acting as molecular “switches” regulate the formation, transport, tethering, and fusion of vesicles transported between organelles (30), which play important roles in the development or virulence of fungal strains, as has been shown in the plant pathogenic fungi *Magnaporthe oryzae* (58, 59) and *Fusarium graminearum* (60). On the other hand, a variety of Rab proteins, such as Rab2, Rab4, Rab5, Rab7, Rab11, and Rab22a, are involved in the regulation of virus replication, transport, assembly, and release (61–66). To verify the hypothesis that hypovirulence caused by CaPV1 was due to interference with vesicle transport of the host fungus, a key Rab gene, *CaRab7*, downregulated in the CaPV1-infected strain was selected, and its mutants were obtained. Mutants showed a reduced growth rate and virulence, indicating that *CaRab7*, the core gene of the vesicle transport system, regulated fungal growth and virulence. Rab7 plays a role in the transformation of early endosomes into late endosomes, mediates the fusion of late endosomes with lysosomes or vacuole membranes (67), and participates in the formation, maturation, and transport of autophagosomes (68–70). A recent study also revealed that Rab7- and retromer-based endolysosomal trafficking play essential roles in effector secretion during host invasion (71). In summary, the hypovirulence caused by CaPV1 might be, at least partially, ascribed to its influence on the vesicle transport system. Meanwhile, this study demonstrates for the first time that particle-encapsulated dsRNA mycovirus could affect host virulence by affecting the host vesicle transport system, which provides new insights for elucidating the interaction mechanism between mycovirus and the host fungus, thereby providing a theoretical basis for the development of the biocontrol potential of mycovirus and resolving the pathogenic mechanism of *Colletotrichum* species.

## MATERIALS AND METHODS

### Strains and growth conditions

The *C. alienum* strain YC-31 was originally isolated from *Camellia oleifera* leaves infected by anthracnose in Hunan Province, China. The *C. fructicola* strains Cf10-6 and LY5-1, *C. spaethianum* strain Cs4-1 and *C. gloeosporioides* strain Cg2-1 were originally isolated from pepper fruits affected by anthracnose, *C. oleifera*, *Paris polyphylla,* and citrus leaves, respectively. All the fungal strains were cultured on potato dextrose agar (PDA: 200 g peeled potato, 20 g dextrose, and 15 g agar in 1 L ddH_2_O) plates at 28°C in the dark. For dsRNA, total RNA and DNA extraction, and conidiation analysis, mycelial plugs were cultured on liquid PDB (potato dextrose broth) at 28°C with orbital shaking at 180 rpm for 4–7 days.

### Viral dsRNA extraction and purification

DsRNAs were extracted from fungal mycelium using CF-11 cellulose (Sigma, St. Louis, MO, USA) column chromatography as described by Morris and Dodds (72) with modifications (72). To eliminate residual DNA and ssRNA, the extractions were digested with DNase I and S1 nuclease (Thermo Fisher Scientific, Waltham, USA). Finally, the extracted dsRNAs were analyzed on a 1% (wt/vol) agarose gel and visualized by a UV transilluminator (ProteinSimple, Silicon Valley, CA, USA) after staining with 0.1 mg/mL Gold View I.

### cDNA cloning, molecular sequencing, and sequence analysis

The cDNA sequences of dsRNAs were determined by Sanger sequencing of amplicons from a cDNA library generated with random hexadeoxynucleotide primers using the method described previously (38). The amplified products were ligated into the pMD18-T vector (TaKaRa, Dalian, China) and transformed into *Escherichia coli*-competent cells. Sequence gaps between clones were filled by RT-PCR using primers designed according to the sequences obtained from the initial round of cDNA sequencing. The terminal sequences of each dsRNA were obtained using a ligase-mediated terminal amplification method. All nucleotide sequences were confirmed by sequencing at least three independent overlapping clones. The full-length viral genome sequences were assembled by DNAMAN. Potential open reading frames (ORFs) were deduced, and their homologous amino acid (aa) sequences were searched in the NCBI web server ORF Finder (https://www.ncbi.nlm.nih.gov/orffinder/) and BLASTp programs (https://blast.ncbi.nlm.nih.gov/Blast.cgi). Conserved domains were identified *via* the CDD database (http://www.ncbi.nlm.nih.gov/Structure/cdd/wrpsb.cgi). Multiple sequence alignments were performed using CLUSTLx with default settings and visualized using GeneDoc. Phylogenetic analysis was carried out with the neighbor-joining (NJ) method in MEGA 7 programs (73). Bootstrap values supporting the phylogenetic tree were calculated after 1,000 resamplings, and the best-fit model of protein substitution was determined in MEGA 7.

### Virion purification and transmission electron microscope observation

The viral particles were purified from the mycelia of *C. alienum* strain YC-31, as previously described with minor modifications (74). The fungus was grown on potato dextrose broth (PD) for 5 days, and mycelia were harvested. Approximately 50 g of mycelia was ground in liquid nitrogen and mixed with 4 volumes of phosphate buffer (0.1 M sodium phosphate, containing 2% Triton X-100, pH 7.0). The mixture was separated by high-speed centrifugation at 12,000 × *g* for 30 min. The supernatant was collected to extract virions by ultracentrifugation with a SW55 rotor in a Beckman at 100,000 × *g* at 4°C for 2 h, and then the sediment was collected. The resultant pellet was resuspended in 0.1 M phosphate buffer, loaded on top of a 20%–50% (wt/vol) sucrose density gradient and ultracentrifuged for 2.5 h at 100,000 × *g*. Each fraction was collected and used for nucleic acid extraction to monitor the presence of viral dsRNAs. The dsRNAs from the viral particles were extracted with phenol-chloroform isoamyl alcohol and detected using 1% agarose gel electrophoresis. Purified viral particles were negatively stained with 1% uranyl acetate and observed under TEM (H7650, Hitachi). The structural proteins from viral particles were detected by sodium dodecyl sulfate (SDS)-polyacrylamide (12%) gel electrophoresis and stained with Coomassie brilliant blue R250.

### Virus curing and viral particle transfection

Protoplast regeneration was used to eliminate CaPV1 from strain YC-31. The preparation of fungal protoplasts was conducted using the method described by Lau et al. (14). The regenerated derivative strains were obtained and individually cultured on PDA plates. dsRNA extraction and RT-PCR were used to investigate the presence or absence of CaPV1 in these derivative strains. The primers used are listed in Table S1.

Protoplast transfection was used to introduce the viral particles of CaPV1 into other virus-free *Colletotrichum* strains by a polyethylene glycol 6000 (PEG 6000)-mediated method, as previously described (43). Protoplasts were adjusted to a concentration of 1 × 10^7^ protoplasts/mL and mixed with purified virus particles. Almost 200 µL of the mixed suspension was evenly spread onto regeneration media (0.7 mol/L sucrose, 0.5 g/L yeast extract, and 15 g/L agar) plates and inoculated at 28°C for 3 days. Singly regenerated colonies were selected and subcultured on fresh PDA and then subjected to detection of CaPV1 by dsRNA extraction and RT-PCR amplification using viral-specific primer pairs.

### Biological testing

The morphological and cultural traits of the different fungal strains used in this study were assessed as previously described (33). Mycelial plugs collected from 4-day-old actively growing plates were placed in the center of fresh PDA plates and incubated for 5 days in darkness at 28°C. Colony diameters were measured to calculate the growth rates, with each strain having at least three replicates. Morphological characteristics, such as colony shape, color, and density, were recorded at 5 days.

For virulence assays, fresh mycelial agar plugs of each strain were inoculated on detached camellia leaves, pear and apple fruits, which were wounded by pin-pricking with a sterilized needle. All inocula were maintained in a humid container at 27°C. The experiments were conducted twice for each treatment, with each treatment having five replicates. Lesions developed on the inoculated leaves or fruits were measured and photographed after 5–10 days of inoculation.

To investigate the conidial production of different fungal strains, mycelial plugs collected from 4-day-old actively growing plates were incubated in PD medium with an orbital shaker at 180 rpm for 5 days. Conidia were harvested by filtration and quantified using a hemocytometer under a light microscope. The morphology of conidia was microscopically observed. Each treatment was repeated three times.

The conidial suspension was collected by filtration with three layers of mirror wiping paper, centrifuged at 6,000 × *g* for 10 min, and washed twice with sterile water. For appressorial formation assays, 10 μL of conidial suspension (1 × 10^5^ conidia/mL) was dropped onto plastic hydrophobic coverslips (Thermo Fisher Scientific, MA, USA) and cultured at 28°C in darkness with 100% relative humidity. The appressorial formation rate was measured by microscopic examination of at least 300 conidia. All experiments were independently repeated three times. After the appressoria was produced from the germinated conidia on plastic hydrophobic coverslips, glycerol solutions of 1 mol/L, 2 mol/L, 3 mol/L, 4 mol/L, and 5 mol/L were individually added to the spore area. After standing for 10 min, the collapse of the appressorium was observed under an optical microscope. One hundred appressoria were counted for each treatment, and each treatment was repeated three times.

### Transmission electron microscopy

The virus-infected and virus-free strains were grown on PDA at 28°C for 5 days. Mycelia of strains were fixed in 2% glutaraldehyde, washed with phosphate buffer, postfixed with 1% osmium tetroxide, dehydrated in a graded series of ethanol solutions, embedded in resin, cut into ultrathin sections, stained, and observed by TEM using a previously described method (33).

### Transcriptome analysis

For transcriptome sequencing, mycelia of the CaPV1-infected strain and its isogenetic CaPV1-free strain were cultured simultaneously on liquid PDB for 5 days as previously described, with three biological replicates for each strain. Mycelia were collected and quickly frozen in liquid nitrogen and stored at −80°C for further use. Total RNA was extracted, and sequencing libraries were generated using the NEBNext Ultra RNA Library Prep Kit for Illumina (NEB, USA) following the manufacturer’s recommendations. The prepared libraries were sequenced on an Illumina HiSeq 2500 platform with 150 bp paired-end reads. Library preparation and Illumina sequencing were performed by Novegene Bioinformatics Technology Co., Ltd. (Beijing, China). Low-quality reads and adapter sequences were removed, and the clean data were assembled and annotated. The gene expression level was calculated by the reads per kilobase per million mapped reads (RPKM) value (75). Differentially expressed genes were selected according to the criteria of *P* value < 0.05 and |log2FoldChange| > 1. Gene ontology (GO) annotations of unigenes were conducted by the program Blast2GO. The Kyoto Encyclopedia of Genes and Genomes database (KEGG) was used to annotate the genome pathways. KEGG pathway enrichment analysis of DEGs was carried out by the R software packages GSEABase and Gostats, and the results were visualized by the R software packages ggplot2 and pheatmap.

To verify the reliability of the transcriptome data, some DEGs were selected for real-time quantitative polymerase chain reaction (qRT-PCR). The primers used are listed in Table S2. Total RNA was extracted and reverse transcribed with a Maxima H Minus First Strand cDNA Synthesis Kit with a dsDNase kit (Thermo Fisher Scientific, MA, USA). The qRT-PCR mixtures were composed of 10 µL of SYBR Green Premix Pro taq HS qPCR kit (AG, Changsha, China), 1 µL of cDNA, 4 pmol primers, and nuclease-free water to a final volume of 20 µL. Amplification conditions were set as follows: 2 min at 95°C, followed by 40 cycles consisting of 15 s at 95°C and 30 s at 60°C. Gene expression was calculated using the 2^−∆∆Ct^ method, and the actin gene was used as an internal reference. For each gene, data were taken from three independent biological replicates.

### Endocytosis assays

We used FM4-64 (Molecular Probes Inc., Eugene, OR, USA) for endocytosis analysis. Fungal strains were grown on CM liquid medium for 16 h at 28°C. FM4-64 was dissolved in distilled water to a final concentration of 5 µg/mL. Mycelia were washed with distilled water, stained with FM4-64 on glass slides, and observed under a fluorescence microscope (Zeiss LSM710, 63 × oil). The filter cube sets were as follows: excitation spectra of 535 ± 20 nm, emission spectra of 610 ± 30 nm, and exposure time of 800 ms.

### Generation of CaRab7 deletion and complementation mutants

To investigate the function of the *CaRab7* gene, *CaRab7* null mutants were generated by vector construction and transformation as previously described (76). For genomic DNA (gDNA) isolation, the mycelia of virus-free strain YC-31-P23 were cultured at 28°C at 180 rpm for 5 days, and gDNA was extracted using the cetyltrimethylammonium bromide (CTAB) procedure. We first amplified the upstream and downstream flanking sequences of the target *CaRab7* gene with the primers CaRab7-SYF/CaRab7-SYR and CaRab7-XYF/CaRab7-XYR, respectively. The upstream and downstream flanking sequences were ligated to plasmid pCX62 containing the hygromycin phosphotransferase gene by enzyme digestion (*Kpn I*, *Hind* III, *BamH I* and *Xba I*; TaKaRa) and ligation reaction (T4 ligase; TaKaRa). The final recombinant plasmid was transferred to *DH5α* for propagation and then transformed into the virus-free strain YC31-P23 by the PEG-mediated protoplast transformation method. Gene knockout mutants were screened with continuous subculture three times on a selective medium, PCR with three sets of PCR primer pairs (CaRab7-LF/HYR, YGF/CaRab7-RR, and CaRab7F/CaRab7R), and qRT‒PCR detection. The primers used for vector construction and gene deletion identification are listed in Tables S3 and S4.

For complementation of the *CaRab7* gene deletion mutant, a DNA fragment of almost 1.5 kb carrying the native promoter and full-length ORF of *CaRab7* was amplified and inserted into the KSTNP expression vector (G418 resistance). After sequencing, the fused KSTNP plasmid was transformed into protoplasts of the Δ*CaRab7* mutant to acquire the complemented strains.

### Statistical analysis

Data on the biological properties were analyzed by SPSS Statistics 21.0 and GraphPad Prism v7.0. Differences between the treatments were compared using one-way analysis of variance (ANOVA), and means were compared using Student’s *t-*test. *, *P* < 0.05; **, *P* < 0.01.

## Data Availability

All the sequencing reads for CaPV1-infected strain YC-31 and its isogenetic CaPV1-free strain were available in the NCBI SRA database (BioProject PRJNA993291). The full-length cDNA sequences for large dsRNA 1 and small dsRNA 2 of the CaPV1 genome have been deposited in GenBank under the accession numbers OR266096 and OR266097, respectively.

## SUPPLEMENTAL MATERIAL

Supplemental Material mbio.02530-23-s0001.docx Tables S1 to S4, Fig. S1 to S3, sequences of the CaPV1 genome, and sequence of the CaRab7 gene.

ASM does not own the copyrights to Supplemental Material that may be linked to, or accessed through, an article. The authors have granted ASM a non-exclusive, world-wide license to publish the Supplemental Material files. Please contact the corresponding author directly for reuse.
