# Supplementary material for: A novel partitivirus conferring hypovirulence by affecting vesicle transport in the fungus Colletotrichum
Source: mBio. 2024 Jan 9;15(2):e02530-23. doi: 10.1128/mbio.02530-23 (PMC10865989; doi:10.1128/mbio.02530-23)
Supplement: Supplemental Material — Tables S1 to S4, Fig. S1 to S3, sequences of the CaPV1 genome, and sequence of the CaRab7 gene. [file mbio.02530-23-s0001.docx]

**Table S1** List of primers used for CaPV1 detection

| Primer name | Primer sequence (5'-3') |
| --- | --- |
| CaPV1-JCF | GATGTTGAACGGGAAGAGTG |
| CaPV1-JCR | TACCGTCTTGTCACATTTCTCA |
| ACTIN-F | ATGTGCAAGGCCGGTTTCG |
| ACTIN-R | GAGTCCTTCTGGCCCATAC |

**Table S2** List of primers used for RT-qPCR

| Primer name | Primer sequence (5'-3') |
| --- | --- |
| ACTIN-F | ATGTGCAAGGCCGGTTTCG |
| ACTIN-R | GAGTCCTTCTGGCCCATAC |
| CGMCC3_g15108F | GCCATCAGGAGGCACTTCAACA |
| CGMCC3_g15108R | GCCCATCGTCATACGTCACCC |
| CGMCC3_g3498F | CATCTACTATGCTCCCGAGTTTC |
| CGMCC3_g3498F | AGTGAGTCAAGGAACGCATC |
| CGMCC3_g4331F | GACCAAGCCCAAGTTCTTCTAC |
| CGMCC3_g4331R | GGCGCTGTTGATGGAGAAA |
| CGMCC3_g8080F | CCTCCACGACCATCTATCT |
| CGMCC3_g8080R | CCGAAGAGTTGTTTAGGGACTT |
| CGMCC3_g8843F | AGCAGTCTACCTCCCAACTA |
| CGMCC3_g8843R | CAATGGCGTTGATGGTGATG |
| CGMCC3_g12494F | GTCACGATCGGCAAGAAGAA |
| CGMCC3_g12494R | CTTCTCCGTGAAGAAGGTCTTG |
| CGMCC3_g13652F | CACAGGACGATGAAGGATCTG |
| CGMCC3_g13652R | CTCCTTCTGGTTGAGGTTACTC |
| CGMCC3_g4991F | GATACCAAACGACACCCTTACCA |
| CGMCC3_g4991R | TGCGGGCACTCCCACAT |
| CGMCC3_g1063F | GCTCACAGAACACCAGATCAA |
| CGMCC3_g1063R | CTCCAGTTGCTTCTTCCGATAA |
| CGMCC3_g9158F | CGATACGCCCTCGCTTTG |
| CGMCC3_g9158R | CGACGAATCCACCGACCAT |
| CGMCC3_g10154F | ATCACGCCGACGCAAAC |
| CGMCC3_g10154R | GGTTCTCGCTGGCAAAGGA |
| CGMCC3_g11467F | TCGGACAACTGGGATTCGG |
| CGMCC3_g11467R | CGGTGATGGAGACTGAGGGAG |
| CGMCC3_g1032F | CAACCGCACATGGAACTTTATC |
| CGMCC3_g1032R | GTCAACCTCGGCGAAGTATT |
| CGMCC3_g6544F(CaRab7-YGDLF) | AGGTCCTTCTCAAGGTCATTATC |
| CGMCC3_g6544R(CaRab7-YGDLR) | GCCTTGTAGCTTGCACTAAAC |
| CGMCC3_g3096F | CGTCATCATCCCTACCAAAGAG |
| CGMCC3_g3096R | TGCTTGTGGACGGAAATCA |
| CGMCC3_g11013F | AAGAAGGTTGAGCACGAGTT |
| CGMCC3_g11013R | GGGATGCCGTTCTTGTACTT |
| CGMCC3_g2333F | GAAGCTGAAGAAGGGCACTAA |
| CGMCC3_g2333R | ATTGTGATGTCGGTTCCAGAG |
| CGMCC3_g3557F | CCGACAATCGAAGATTCCTACC |
| CGMCC3_g3557R | CTCATGTACTGCTCTCGCATAG |
| CGMCC3_g14875F | GGCACAAGCATCTCCATACA |
| CGMCC3_g14875R | CAGAGGTGTCAAACTCCATCAA |
| CGMCC3_g8077F | CACCATCACCTCCTCCTACTA |
| CGMCC3_g8077R | GCGTATCTGTCAATCTCCTGAA |
| CGMCC3_g7492F | GTCAAGGGTATCCTGCTGTATG |
| CGMCC3_g7492R | GAACTTCGGGACCGTTGATAA |
| CGMCC3_g5147F | TCGAGACCGTCGAGTACAA |
| CGMCC3_g5147R | CCTGGGTGTTCTGGAAGTAATG |
| CGMCC3_g11129F | GAAGTTCGAGTCTGAGCATACC |
| CGMCC3_g11129R | CCTTATCCTGGCTCTTCTTTCC |
| CGMCC3_g5063F | CCGACTACTACGAGGACAAGA |
| CGMCC3_g5063R | GACGGAGTGACCAATGTAGATG |
| CGMCC3_g12847F | TCAAGGAGTACCTCACCAAGTA |
| CGMCC3_g12847R | GGTCTTGTGCAGGACAATCT |
| CGMCC3_g13010F | GTGATGCGGGAGAACATCAA |
| CGMCC3_g13010R | CCAGGTTGTCGGTCTTATCTTG |
| CGMCC3_g9379F | ACGATGTTGTGGAGGACTTG |
| CGMCC3_g9379R | GATGAGGTCGTAGCAAGCATAG |
| CGMCC3_g10737F | CAGGACACCGAAGTTGTTACT |
| CGMCC3_g10737R | CTGTCCCTGGCGAATGATAAA |
| CGMCC3_g6220F | ACGACCGAAGCAAGAGATTAC |
| CGMCC3_g6220R | GTTTGTCTGGCACCGATACT |
| CGMCC3_g2480F | CCCTCAAGACGACCAACAA |
| CGMCC3_g2480R | TCCATCAAATCCGCCATCTC |
| CGMCC3_g5214F | GCCGATATCAGGGTGAAGATAC |
| CGMCC3_g5214R | GCCGATGGCACAAATAACATAG |
| CGMCC3_g8173F | CCTCAGCGAGTACAACAAGAA |
| CGMCC3_g8173R | CGGCTTTGAAGTTGGCAATC |
| CGMCC3_g6793F | GCGAACCCGAAGTCAAATAAAG |
| CGMCC3_g6793R | CTCGCGGATAGAATCTCGAATAG |
| CGMCC3_g10217F | CACCACCGCCTCTGATTATT |
| CGMCC3_g10217R | AACATCCCATCTCTTCCCATTT |

**Table S3** List of primers used for vector construction

| Primer name | Primer sequence (5'-3') | Annotation |
| --- | --- | --- |
| CaRab7-SYF | CGGggtaccCTACGGCGCATTTCAGACC | underline *Kpn* I |
| CaRab7-SYR | CCCaagcttTCGGGTCCGCTATGTATGG | underline *Hind* III |
| CaRab7-XYF | CGCggatccGTTGAGCGAGCAGAGCATC | underline *BamH* I |
| CaRab7-XYR | GCtctagaGCCGTTCCGTTGACTTGA | underline *Xba* I |
| CaRab7-CF | GGTACCCGGGggatcc GAGGATCTCGTCAAGTGTT | underline *BamH* I |
| CaRab7-CR | AACGTTAAGTggatcc TTAGCAGGCGCAGCCGTCC |  |

**Table S4** List of primers for detection of the *CaRab7* mutants

| Primer name | Primer sequence (5'-3') | Annotation |
| --- | --- | --- |
| CaRab7F | GGTCCTTCTCAAGGTCATTATCC | *CaRab7* gene |
| CaRab7R | CTCTCTTGCTCTCCTCAACATC |  |
| CaRab7-LF | CGCATTTCAGACCGAAGGT | the upstream flanker sequences of *CaRab7* gene and some Hyg gene fusion fragments |
| HYR | GCTCCATACAAGCCAACCAC |  |
| YGF | TCGTTATGTTTATCGGCACTTT | the downstream flanker sequences of *CaRab7* gene and some Hyg gene fusion fragments |
| CaRab7-RR | GCCGTTCCGTTGACTTGACT |  |

Fig.S1


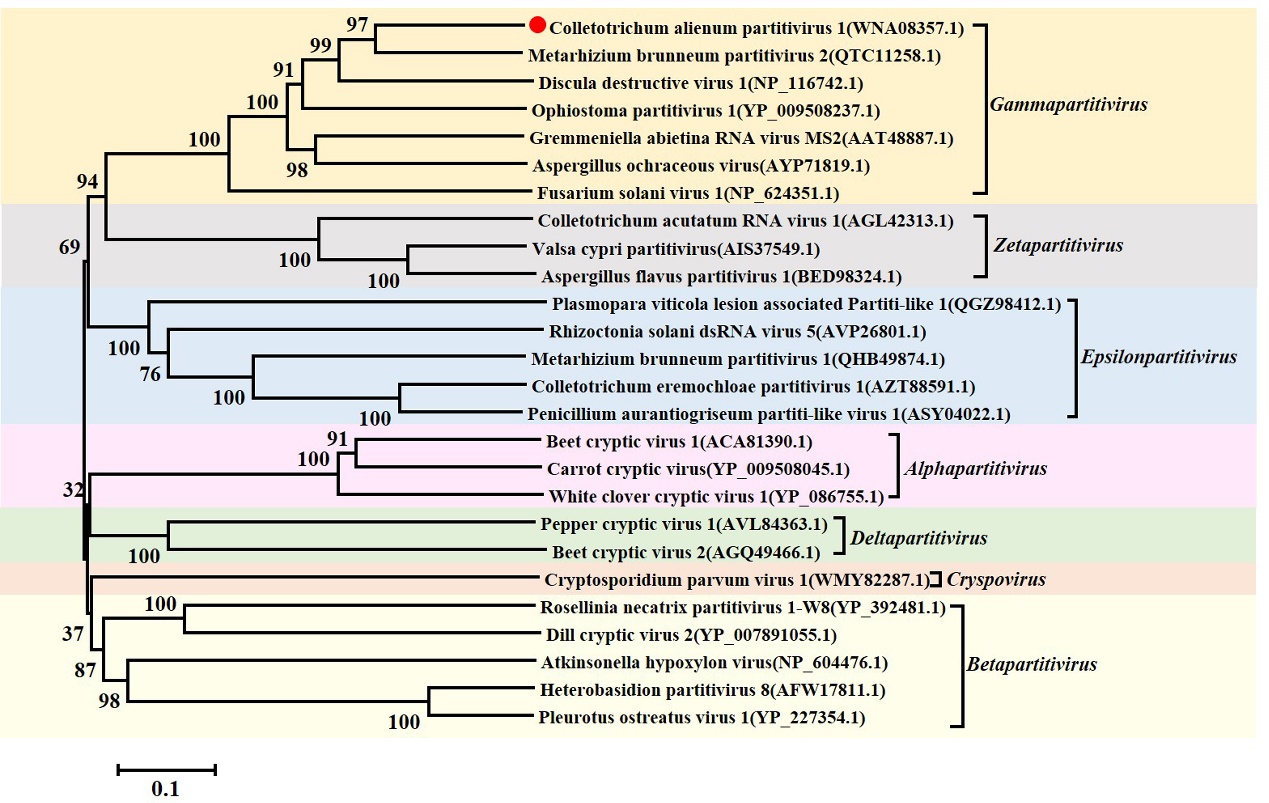


**Fig.S1**. Phylogenetic tree constructed based on the amino acid sequences of the capsid proteins. The GenBank accession numbers of amino acid sequences used for phylogenetic analysis were given in the parentheses. Numbers on the branch nodes indicated bootstrap values calculated after 1000 replications.

Fig.S2


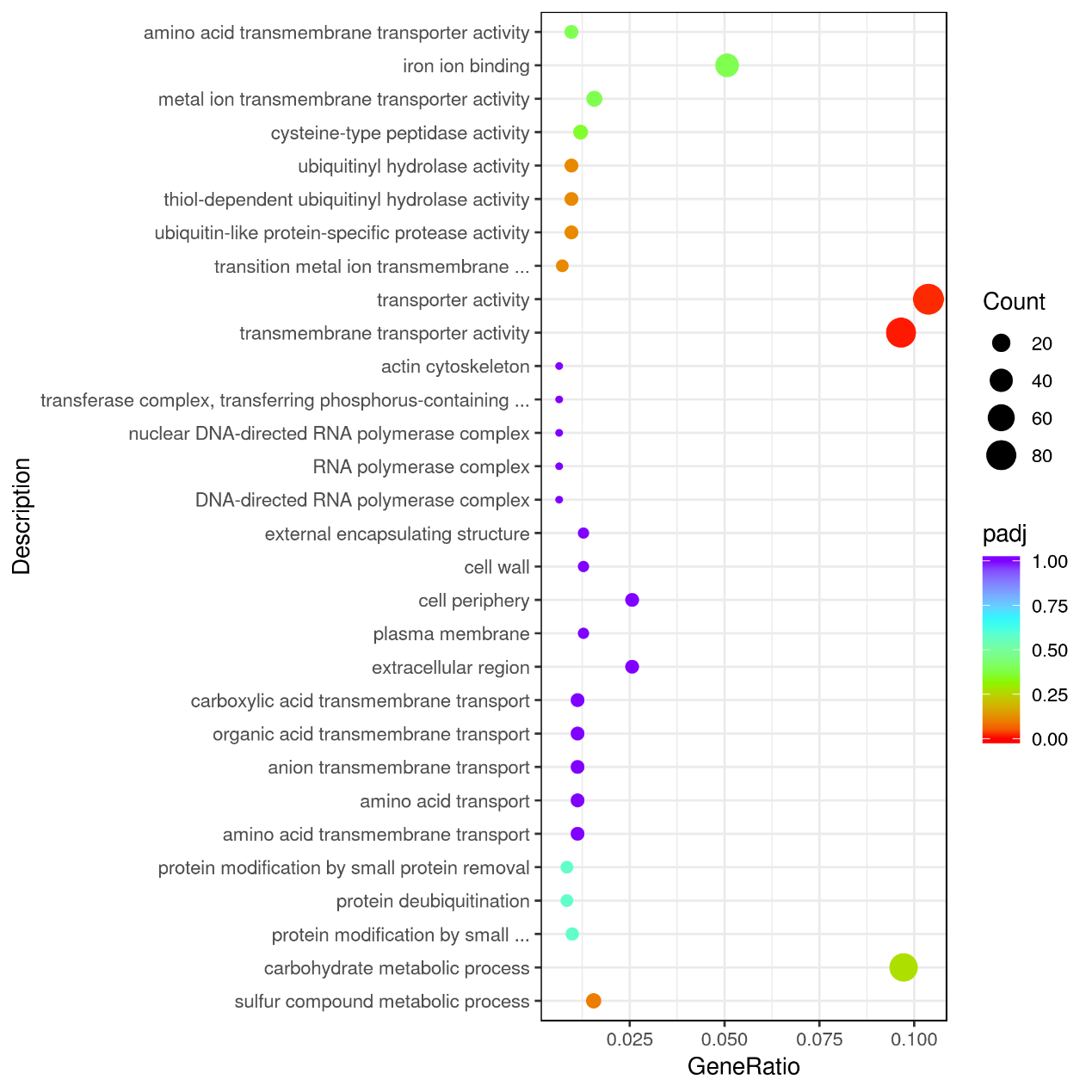


**Fig.S2**. Bubble diagram of the GO enrichment analysis of differentially expressed genes (DEGs) in the biological process, molecular function, and cellular component categories.

Fig.S3


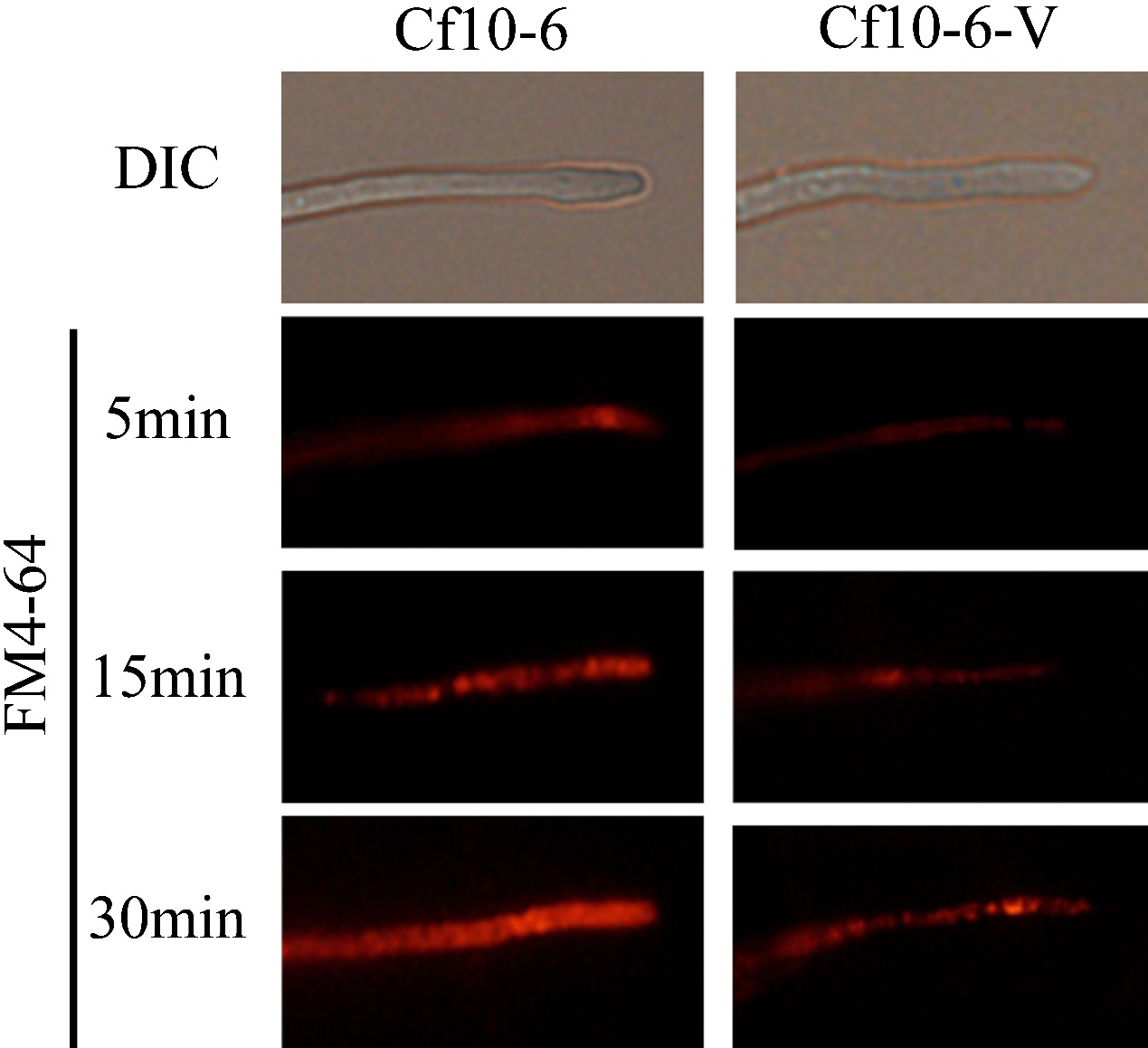


**Fig.S3**. Effect of CaPV1 on endocytosis of *Colletotrichum fructicola* by staining with FM4-64. Time course images of FM4-64 uptake at the hyphal tips were captured using fluorescence microscopy at intervals of 5 minutes, 15 minutes, and 30 minutes. The absorption and internalization of dyes were assessed by observing the presence of red fluorescence.

Sequences of the CaPV1 genome

>dsRNA1

TCGCAAAAGCCTCGAGCTTTTACCAGTTTATCTGGGTTTTGCCTCCTTCACTTCTTGAGCGAAAATGGAAGATTTTACTCAAGATCCAACACAACACTACGTCCTAGCTAAGGGGTCTCATTTGATTGACGCACTCCACCTCCGACCTGCAAAGTCGGGGTCAACCACTAGCGAAGATGTTATCCCTTCCAACTTTGAGGGTCCCAACTTAGTCGAAATAGCTAAGTATGGGGGGTACTCAACATACATGTCTAACTCGAACACCGACGGTTGGGTCCGAGAGACGTTAAAACTGAACGACCGAGAACTTTACGAACAAATCCGTGGATACACCCGCCGACCTCAAGGTACTCCAGGCATGTACAGTGCTCTGAAGAAGTTTGATGGCGAAAAGTGTCCATTCGGAAACCTAGCCCCTTGGCAAAAACGTTGCATGCGTAAAGCGATCAAGAAAGCCTTTAAAGCTTTCAAACTTCCATACAAACGAGAACCTCTCGATTGGCATGAAGTAGGACAGTACCTGAGGCGTGATACGTCAGCAGGATCCACTTTCATGGGCCAAAAGAAGGGTGACGTGATGGAGGAGATTTACCATGAGGCGAGGTGGTTAGGACACCGGATGAAACAGGATGGAAAACGAAAGTTCGATCCAACCAAGATGAGGTTTCCCCCTTGCTTGGCCGGTCAGCGTGGCGGAATGTCCGAGAGAGATGATCCGAAAACGCGCCTGGTGTGGATTTACCCCGCAGAGATGTTGGTTGTCGAGGGATTCTACGCCCCTCGTATGTATCGTGACTTTATGAACGATCCCAATTCGCCGATGTTGAACGGGAAGAGTGCGCAGCGTCTGTACACCGAATGGTGCTGCAACTTGAGGGAAGGAGAAACGTTGTATGGGCTTGACTTCAGCTCTTTTGACACTAAAGTGCCAGCGTGGCTAATACGTGTTGCCTTTTCGATCTTACGTCAGAATATCAATTTTGAGACGTTTGATGGCAAACCTGTGGGTAAACAGGATGCTCAGAAATGGCGTAACGTCTGGGATGCTATGATTTGGTACTTTATCAACACTCCTATTCTTATGCCGGATGGACGAATGTTCAGGAAGCTCCGGGGTGTACCTTCCGGATCCTGGTGGACGCAGATGATCGATTCCGTCGTCAATTACATTTTGATTGATTATCTTGCGAACTGCCAACAGGTAGAGATCCGAAACCTGAAGGTGTTGGGTGATGATAGTGCTGCGAGATCCAATGGCGAGTTTGATCTTGACGTGGCGCAGCAGGATTGCGAACCCTTAGGTATGATACTTAAACCTGAGAAATGTGACAAGACGGTAGACCCAGCCGAATTTAAACTTCTTGGCACTAAGTATCGTGATGGACATGTCCATCGACCGACGGAGGAGTGGTTCAAGCTAGCATTGTACCCAGAGTCAAGCGTGTTTACGCTTGATATTTCTTTCACACGCCTGATAGGCCTGTGGTTAGGTGGCGCGATGTGGGATCGAAAGTTCTGTGAATACATGGACTTTTTCCAGAGTAGCTATCCGTGTCCTGAGGAGGGGTGGTTCTCCAAAGATCAGAAACGGTGGCTTGAGGTCATCTATTCAGGCAAGGCGCCAAGAGGCTGGACTACGAAACGTAGTCTGTTTTGGCGCTCAATCTTCTATACCTATGGTTAGGAGTGTAGTCGTACCGCTGAGGTGCGCGTTTCCGTGTATACGGGGATTCTAACGTGTCGTGTAAACGACCGTGTTAACAAAATCCA

>dsRNA2

TCGCAAAAGCCTCGTCTTTTACTAGTGTATACTGGGTTTTCTCTTGATTTTAGCTCTCTCACTCTTGACTAAAATCATTGCCTCCTTCACCTCTGGACCTAGCTGGCTGCTCGTTCAGATAATGGAATCTTCCGTCGCCCCGAGTGATTCTGCCTCCGCTGCCGGAAGCAAGAAGTCTCGCCCTGGTCGCGCCGAGCGTGCTGCTCGTCGTGCCGCTACCGGATCAATCGCCGGTCAGCCCGCCTCATCCGCCAAGGCCATGACTTTTGCAACCATGACTTCAGCTCCGAAGCCCCAACCGGGTAAATTTCCGATCGTCTTTCAGACCGGTGCCGGTGAACCTTCGCGAGACGTCAACTTCGCTGTCGAGCCCAAAGTGCTCGCTACTTCGCTTTCTACGTTCGTACCTGCCTTTAAGGAGAACCCGAAGTACGCTGAGTTCCTCACTTGGACTGAATACAGCGACGTGGACTTTGAGAAGCAGCTCAAGGTCGCTGCACTTCTGCGCTTGGCCCAGCAGATTGTGCATTCTCACGTGAACATGGGTCTACCTCTTGGCGATTTTGCTCCTGTGAATTCGACCGAAGTCCGTGTACCGGCATCGGTTTCCGCATTCCTCACCCAGTATGGTGAGTTTTCTGTTCCAGCCCTTGGAACCAGATACTTACTTAAAGACTATCCGTCTACCGTGAAGTCTTTGGTCTGGGCGGCCAGTCAACTGTCTGGCTCGCGGTCTCGAAACGGGGTGGTCGAACGTTCATGGCTTCCAGTGTCTGGAAGTGACGGCCATACCAAGCAATTTCTCGCCCATCGGCTTAATGCTTTCCTTAAACAAGCCGATGTTGTGTACTCGTCAACGGTTCTGGAAGATGCCGTGTTATCGGGTACACCCCCAGAGCATTGGAGCGACATCAAGTCAATCTTTGGTGATACGGATGCCGAGCGAGATCGTTTCGACTTTCTCTTCCGTACTTACACCTCTGCTCCACTGTTCGTAACTTCCTTCACCCAGACTCAGTCTCTGTTGGTACTGGAGGAGTTGGGGATTCCCTGGGAGGTACCGTCCGCGGGTCACGTGGATTGGACCTTCAACGTCAAGGAGTTGTTTACCACCCTTGCCGACACCTGGGCTCAAAAGTCGGCGGCATACGCTCAGTTCTTTGAGATGTCGTCGAGCCAGGCCAACAAGATGGCTGCCACTGGCTCTCAGTCGCAGTTGGCGTCTGTGTCTACCACGGACTCAATCACCATCATCAAGACCCATTTGGCATTGTCGGCTCCAGAGTTTTCTTTGGTGGCGTGCTTTCCTGCAACCGGTGTCTTTTCCGGACAGCAGACTCAGAATGTTGTTCTGACGACACCTCTCTCTGTGAAACAGAGAGCCACAGAGTTCATACAGATGGATTGGCGTTAACCAATGACGATCAATTTCATCTGTCTTTCTCTATTTCAGTCGTCTGACGGTGGGCTTTCGAACCCCCACTGTGTAAATAACTAAATCTGAAAAACACCTGCGTGTAAACGCGGAAACCCAGTGTAGACTGGTCACATTTGTTTTAACAAAACGGTGTGGAAACGCACTCGTGTATACGAGAGGCGTGACCAATAAAATCCA

Sequence of the *CaRab7* gene

> CaRab7

ATGTCATCGAGAAAGAAGGTCCTTCTCAAGGTGGGCTTTATCGGCGGCCCCAAACACACGTGCGGCATAATTGATCGTTTCTGTGTTGCAGGTCATTATCCTTGGCGATAGCGGTGTCGGCAAGACGAGTTTGATGAACCAATATGTATGTTTTCTTGCTGCGCTGTCTGGCGGCCCGACCGGTGGTAGCCTAGAGTACTGATTTGGCTATTTCTACCTTTTAGGTCAACAAGAAGTTTAGTGCAAGCTACAAGGCCACGATCGGCGCCGACTTCCTGACCCGCGAGGTCCTCGTCGATGACCGGCAGGTGACGATGCAGGTGGGTGACGCCGCGAACACATTGCCGATGGAAACAACGAACGCGTTGCTGACGGACAATAGCTCTGGGACACTGCGGGACAGGAGCGCTTCCAGTCGCTGGGTGTCGCCTTTTACCGCGGAGCTGACTGCTGCGTACTCGTCTACGACGTCAACAACTCCAAGAGCTTCGATGCTCTGGACAGCTGGAGGGACGAGTTCCTGATCCAGGCTTCTCCGCGTGACCCCGACAACTTCCCATTCGTCAGTATCTTGAAGTCCTAGAGGGATAGGACATGGACACTGATAAGAGATTAGGTCGTCCTTGGAAACAAGATTGATGTTGAGGAGAGCAAGAGAGTTGTAAGTTTTGGTGTCACCTGCGGCTACCCGACGACAAGCTTACCATTTGATTAGATCTCGACCAAGCGTGCGATGACCTTCTGCCAGTCCAAGGGCGGGATTCCCTACTTCGAGACCAGTGCGAAGGAGGCTATCAACGTCGAGCAGGCTTTTGAAGGTTGGTTGCCTCTTCCAAGGTGAAATCTTAGAGATGCTAACATTGTTCACCAGTCATTGCACGAAATGCCCTTGCTCAGGAGGAGTCGGAAGAGTTCAGCGGGGACTACCAGGATGTCATCAACATTCCCATTGAGAACCCCCGGGACGGCTGCGCCTGCTAA
